# Hsa-miR-183-5p Modulates Cell Adhesion by Repression of *ITGB1* Expression in Prostate Cancer

**DOI:** 10.3390/ncrna8010011

**Published:** 2022-01-18

**Authors:** Carolina Oliveira-Rizzo, María Carolina Ottati, Rafael Sebastián Fort, Santiago Chavez, Juan Manuel Trinidad, Andrés DiPaolo, Beatriz Garat, José Roberto Sotelo-Silveira, María Ana Duhagon

**Affiliations:** 1 Laboratorio de Interacciones Moleculares, Facultad de Ciencias, Universidad de la República, Iguá 4225, Montevideo 11400, Uruguay; coliveira@fcien.edu.uy (C.O.-R.); mcottati@gmail.com (M.C.O.); rfort@fcien.edu.uy (R.S.F.); schavez@fcien.edu.uy (S.C.); jtrinidad@fcien.edu.uy (J.M.T.); bgarat@fcien.edu.uy (B.G.); 2 Departamento de Genética, Facultad de Medicina, Universidad de la República, Av. Gral. Flores 2125, Montevideo 11800, Uruguay; 3 Departamento de Genómica, Instituto de Investigaciones Biológicas Clemente Estable, Montevideo 11600, Uruguay; apdipaolo@gmail.com (A.D.); sotelojos@gmail.com (J.R.S.-S.); 4 Departamento de Biología Celular, Facultad de Ciencias, Universidad de la República, Iguá 4225, Montevideo 11400, Uruguay

**Keywords:** cancer, microRNA, focal adhesion, miR-183, prostate, ITGB1, TCGA, AGO-PAR-CLIP

## Abstract

Prostate cancer is a major health problem worldwide. MiR-183 is an oncomiR and a candidate biomarker in prostate cancer, affecting various pathways responsible for disease initiation and progression. We sought to discover the most relevant processes controlled by miR-183 through an unbiased transcriptomic approach using prostate cell lines and patient tissues to identify miR-183 responsive genes and pathways. Gain of function experiments, reporter gene assays, and transcript and protein measurements were conducted to validate predicted functional effects and protein mediators. A total of 135 candidate miR-183 target genes overrepresenting cell adhesion terms were inferred from the integrated transcriptomic analysis. Cell attachment, spreading assays and focal adhesion quantification of miR-183-overexpressing cells confirmed the predicted reduction in cell adhesion. *ITGB1* was validated as a major target of repression by miR-183 as well as a mediator of cell adhesion in response to miR-183. The reporter gene assay and PAR-CLIP read mapping suggest that ITGB1 may be a direct target of miR-183. The negative correlation between miR-183 and *ITGB1* expression in prostate cancer cohorts supports their interaction in the clinical set. Overall, cell adhesion was uncovered as a major pathway controlled by miR-183 in prostate cancer, and ITGB1 was identified as a relevant mediator of this effect.

## 1. Introduction

Prostate cancer (PrCa) is the second most frequently diagnosed cancer and the fifth leading cause of cancer-related deaths in men worldwide [1]. Although the disease frequently evolves slowly, a minority of patients will progress to an aggressive form that is resistant to androgen deprivation therapy. Despite the international efforts to improve diagnosis and treatment, no significant reduction in the mortality rate has been reached.

MicroRNAs (miRNAs) are endogenous small non-coding RNAs (19–22 nt) that are able to modulate protein levels through their sequence-specific interactions with complementary target mRNAs [2,3]. miRNA regulation has a wide impact in human gene expression [2,4] and is extensively altered in cancer, including PrCa [5,6,7,8,9]. Individual miRNAs usually target several mRNAs [10], affecting diverse cellular pathways, thus contributing to multiple cancer hallmarks. Although miRNA-target studies are vast, a complete picture of the effect of a given miRNA with an appropriate hierarchization of its pleiotropic effect is still scarce.

Hsa-miR-183-5p.1 (5′-UAUGGCACUGGUAGAAUUCACU-3′), hereinafter referred to as miR-183, is coded by a member of a highly conserved gene cluster located at the 7q31-34 locus of the human chromosome [11]. Several studies have indicated that miR-183 is abnormally expressed in many tumors [12], showing both oncogenic and tumor suppressor functions, depending on the tissue type. In PrCa, miR-183 has consistently exhibited an oncogenic role, and its overexpression has been validated by mounting PrCa miRNA profiling studies [13,14,15,16,17,18,19,20,21,22]. Additionally, the expression of miR-183 in biopsies has been associated with disease status, diagnosis and prognosis in PrCa [13,14,16,22,23,24]. Ueno et al., have proposed that the inhibition of miR-183 reduces cell proliferation and migration by up-regulating *DKK-3* and *SMAD4* via WNT/β-catenin signaling [14]. These cellular effects were later confirmed by Dai et al., who identified TPM1 as another direct target of miR-183. The authors also proposed that exosomal-loaded miR-183 can provoke a similar regulation in neighboring cells through paracrine signaling [25]. On the other hand, the overexpression of the miR-183 full cluster was shown to regulate zinc homeostasis by diminishing the labile zinc pool and reducing zinc uptake and to directly repress *hZIP1* transcript and protein through miR-182 and miR-96 repressive interaction (not mir-183) [21,26]. Since the decrease in zinc levels promotes disease progression, the study supports an oncogenic function of the cluster in PrCa. More recently, candidate tumor suppressor long non-coding RNAs (lncRNAs) were demonstrated to interact with miR-183 and compete for its target mRNAs binding in PrCa. Specifically, *CASC2* competes with *SPRY2* for miR-183 binding to rescue the expression of SPRY2 in PrCa cells, thus enhancing the sensitivity of PrCa cells to docetaxel through the ERK signaling pathway [19]. Additionally, *LSAMP-AS1* competitively binds to miR-183, inhibiting epithelial–mesenchymal transition (EMT), proliferation, migration and invasion, and the direct repression of the tumor suppressor *DCN* (Decorin) is proposed as a mediator of this phenotype [20]. Interestingly, miR-183 positively regulates the PSA level in PrCa cells and serum by direct interaction with PSA mRNA [27]. The robust and pleiotropic impact of miR-183 in PrCa suggest it could be a major miRNA driver of the disease and, thus, guarantees further investigation of its contribution to disease initiation and progression. 

The aim of our study was to identify novel mechanisms of miR-183 action in PrCa, discovered from hypothesis-unbiased transcriptomic studies, to uncover the major relevant pathways modulated by miR-183 in the disease. We first analyzed clinical samples and confirmed that the expression of miR-183 is increased in metastasis and tumor compared to normal prostate tissue and is associated with worse clinical features. An integrated analysis of cell line and tissue transcriptomic data identified 135 candidate target genes that were enriched in cell adhesion pathways. We demonstrated that miR-183 modulates the adhesion of prostate cell lines and identified *ITGB1* as a target gene contributing to this phenotype. *ITGB1* is downregulated in PrCa samples and negatively correlates with miR-183 in PrCa tissue of the PRAD-TCGA cohort. Furthermore, we demonstrated that miR-183 reduces the number of focal adhesions, the main surface complexes in which ITGB1 mediates adhesion to the extracellular matrix (ECM). These results lead to the proposal that cell adhesion is a major prostate cancer hallmark modulated by miR-183, and *ITGB1* is a target of miR-183 repression in PrCa, contributing to this phenotype in vitro and possibly in the clinical set.

## 2. Results

### 2.1. Expression of MiR-183 Is Increased in Tumor Tissue and Associates with Worse Clinical Features in PrCa

MiR-183 upregulation in neoplastic tissue compared to normal tissue has been identified in the majority of PrCa miRNA studies, including the two largest published cohorts from the Memorial Sloan-Kettering Cancer Center (MSKCC) [28] and The Cancer Genome Atlas (PRAD-TCGA). We first measured the expression of miR-183 in tumor and normal matched adjacent tissue from radical prostatectomies of seven Uruguayan patients by RT-qPCR, which confirmed the significant upregulation of miR-183 in this cohort (*p*-value = 0.0469) and suggested its association with the tumor histological grade (Supplementary Figure S1). Furthermore, since the association between miR-183 expression and patient clinical parameters has not been thoroughly studied and new clinical data has become available, we re-analyzed the expression level of miR-183 in the MSKCC and PRAD-TCGA samples. The samples were classified by the status of the individual clinical variables and the expression level of miR-183 was compared between groups. Positive associations between miR-183 expression and PSA level at diagnosis and prior to radical prostatectomy and shorter time until recurrence were found in the MSKCC cohort (Table 1). Biopsy and pathologic Gleason Score, clinic and pathologic T, overall survival (month) and lymph node involvement were also analyzed but did not yield significant associations with miR-183 levels (Supplementary Figure S2). We also found that the metastatic tissues had a higher expression of miR-183 than their normal counterparts in that cohort. The analysis of the PRAD-TCGA samples yielded a positive association between miR-183 expression and biochemical recurrence, clinical T stage, Gleason Score and pathologic N status (Table 1), while there were no significant associations with first biochemical recurrence, PSA or residual tumor (Supplementary Figure S2). Although Li et al. reported similar results for pathologic T, clinical M status and overall survival [13], we found a less significant association between miR-183 expression and clinical M status (M1 vs. M0) (*p*-value = 0.0735) and no significant association with patient survival using OncomiR statistics [29] (Supplementary Figure S2). Altogether, these findings support the oncogenic role of miR-183 and its association with disease evolution in PrCa.

### 2.2. Direct Candidate Target Genes of MiR-183 Are Related to Cell Adhesion

**Figure 1:**
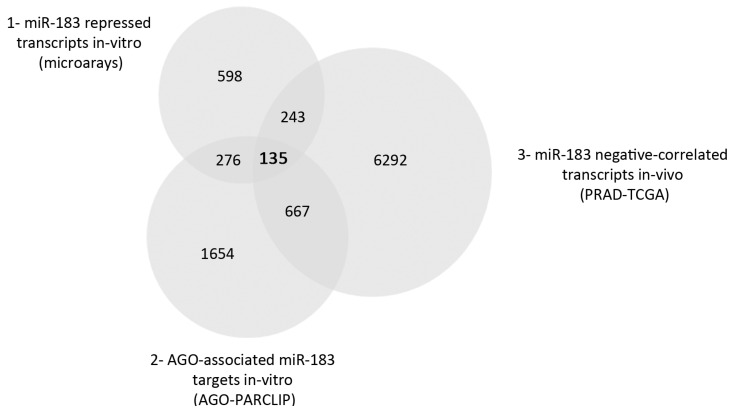
Venn diagram of the three gene lists used for the selection of miR-183 candidate direct target genes. 1: Genes downregulated and upregulated upon transfection of miR-183 mimic or inhibitor, respectively, identified by gene expression microarrays of the DU145 and LNCaP cell lines (fold change ≥ 1.25 in at least two of the four conditions assessed). 2: Argonaut-associated transcripts containing the 6-mer (2–7 nt) sequence complementary to the miR-183 seed extracted from prostate cell lines in the AGO-PAR-CLIP experiments performed by Hamilton et al. [31] (at least one read in three out of five cell lines published). 3: Transcripts showing a negative Spearman correlation with miR-183 expression (*p*-value < 0.05, r^2^ < 0) in the PRAD-TCGA samples.

To elucidate the major molecular mechanisms affected by miR-183 upregulation in PrCa, we analyzed PrCa transcriptomes and performed in silico predictions to select candidate target genes directly regulated by miR-183. First, we carried out total mRNA profiling experiments using Affymetrix microarrays of DU145 and LNCaP cell lines 24 h after the transient transfection of an miR-183 mimic, miR-183 inhibitor or a control RNA. Since miR-183 in PrCa has not been linked to androgen signaling directly, we chose these two cell lines as models of the androgen-sensitive and insensitive PrCa. The modulation of miR-183 expression upon transfection was confirmed by qRT-PCR. A significant increase in the miR-183 level in the mimic transfectants was detected in the DU145 and LNCaP cell lines, while a reduction of the levels of miR-183 was detected after the transfection of the miR-183 inhibitor molecule in the LNCaP cells but not in the DU145 cells (Supplementary Figure S3). Since the effect of miR-inhibitors on the stability of their endogenous miRNA targets is not completely understood, a repressive effect without a reduction in the miRNA level cannot be ruled out [30]; therefore, we included the inhibitor experiments in the transcriptomic analysis. The comparison of the miR-183 mimic and inhibitor experiments of both analyzed cell lines resulted in 1252 genes that were inversely correlated to miR-183 modulation (fold change ≥ 1.25), i.e., downregulated after miR-183 mimic transfection and upregulated after miR-183 inhibitor transfection (Supplementary Table S1). To identify putative direct target genes of miR-183 that are active in the miRNA pathway in the prostate tissue context, we analyzed the AGO-PAR-CLIP experiments of prostate cell lines published by Hamilton et al. [31]. From this dataset, we selected 2732 argonaut-associated transcripts containing motifs perfectly complementary to the 6-mer (2–7 nt) miR-183 seed (Supplementary Table S2), thus including all the canonical seed types [32]. Finally, to incorporate an in vivo target selection criterion, we chose 7337 transcripts from the PRAD-TCGA RNA-seq dataset showing a significant negative Spearman correlation with miR-183 expression (r^2^ < 0, *p*-value < 0.05) (Supplementary Table S3). The thresholds chosen for the three gene lists were set to be of low stringency, considering the concerns about the caveats in miRNA methods and interpretation that have been raised in the literature [30,33,34,35,36] and thinking of this analysis as a discovery approach. The intersection of the 3 gene lists yielded 135 possible direct targets (Supplementary Table S4) of repression by miR-183 in prostate cells (Figure 1).

To investigate the function of the 135 direct miR-183 candidate targets genes, Wikipathways, Kyoto Encyclopedia of Genes and Genomes pathways (KEGG) and Gene Ontology (GO) Cellular Component were studied using the Enrichr online tool [37,38]. Cellular processes related to cell adhesion, such as focal adhesion, proteoglycans, cell migration, mesenchymal–epithelial transition (MET) and regulation of actin cytoskeleton appeared to be overrepresented, whereas focal adhesion was the unique significantly overrepresented GO Cellular Component (*p*-value ≤ 0.001) (Table 2).

### 2.3. Overexpression of MiR-183 in Prostate Cells Causes a Decrease in Cell Adhesion In Vitro

**Figure 2:**
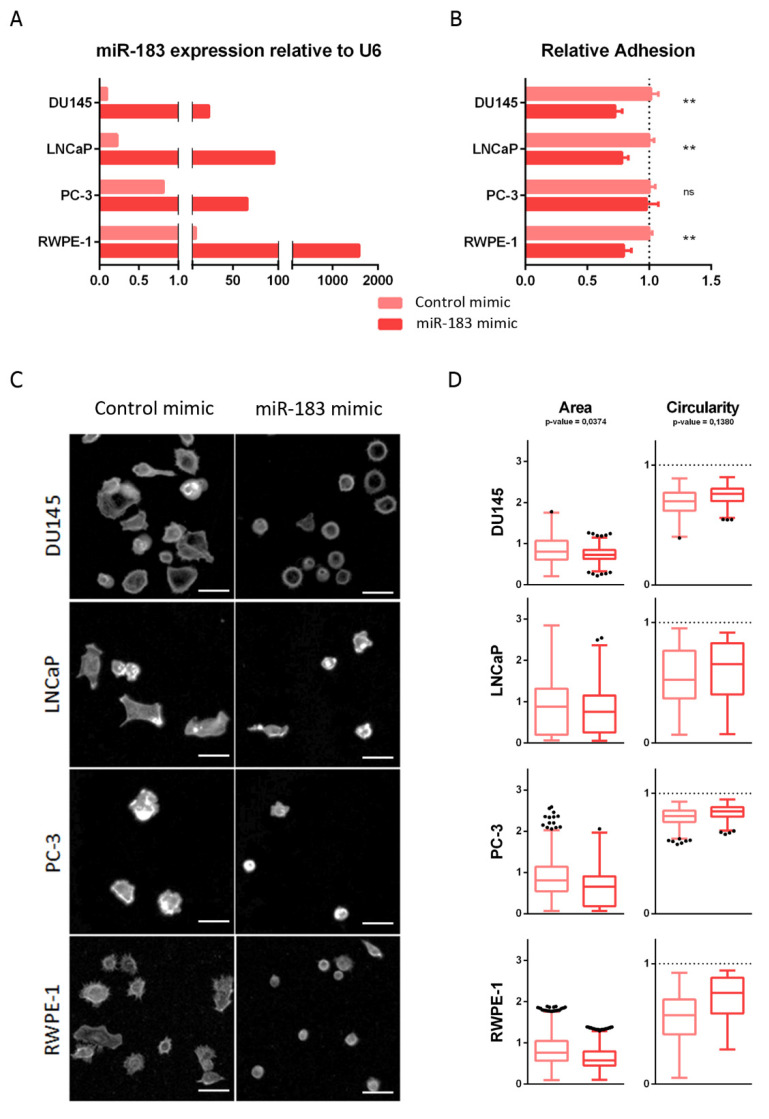
Effect of miR-183 on cell adhesion. (**A**) RT-qPCR measurement of miR-183 relative to RNAU6 (2^−∆∆CT^) 72 h after transfection of 20 nM miR-183 mimic or control RNAs in the indicated cell lines. (**B**) For the cell adhesion assay, the cells were harvested 72 h after transfection and seeded on cell culture surface-treated polystyrene 96-well plates, then allowed to adhere for 0.5 or 1 h (depending on the cell line), and the non-adherent cells were removed. Initial and remaining (attached) cells were quantified in independent wells using CellTiter-Glo (Promega). The fraction of attached cells in the miR-183 mimic relative to the control is shown as the mean ± standard deviation from at least three independent experiments. An unpaired t-test was applied to assess the statistical significance of the difference between conditions. (**C**) 72 h after transfection, the cells were harvested, plated on coverslips and allowed to spread for 3 h. The cells were then fixed, permeabilized and stained with Alexa Fluor 488-conjugated phalloidin to reveal the actin cytoskeleton. Images were captured with a Fluorescent Cell Imager using a monochrome camera and a magnification of 175×. Scale bars show 25 μm. Representative images are shown. (**D**) Cell area and circularity measured in cell adhesion assays of B were retrieved using ImageJ Software. Approximately 300 individual cells were analyzed for each cell line. A paired *t*-test was performed, considering the four cell lines as biological replicates, to assess the statistical significance between conditions. ** *p*-value < 0.1, ns = not significant.

The enrichment of focal adhesion function among the miR-183 selected candidate direct target genes led us to assess its effect on cell adhesion in vitro. First, we analyzed the effect of miR-183 on the adhesion to standardly treated cell culture plates (see Section 4.7), after trypsinization of the monolayered culture, using normal (RWPE-1) and malignant (DU145, LNCaP and PC-3) prostate cell lines. For this purpose, the overexpression of miR-183 was forced by the transient transfection of mimic or control RNAs and, 72 h afterwards, the adhesion of cells to the plate surface was compared. The modulation of miR-183 expression after transfection was confirmed by qRT-PCR (Figure 2A). Quantitatively, miR-183-overexpressing DU145, LNCaP and RWPE-1 cells showed statistically significant decreases in adhesion, to 0.73 ± 0.06 (27%), 0,78 ± 0.05 (22%) and 0.79 ± 0.06 (21%), respectively, relative to the control cells (*p*-value < 0.01), while overexpressing PC-3 cells showed no significant change in adhesion relative to control cells in this experimental condition (Figure 2B). Since adhesion is a complex process, involving not only cell receptors binding to substrate ligands but also the modulation of intracellular signaling and the cytoskeleton, we sought to analyze the morphological spreading during adhesion by microscopic visualization of the actin cytoskeleton using phalloidin staining (Figure 2C). After 3 h of seeding, the overexpression of miR-183 produced a decrease in the area (*p*-value = 0.0374) and a tendency to increase the circularity (*p*-value = 0.1380) of the cells relative to the control transfected cells (Figure 2D). Therefore, both the quantitative and qualitative results were consistent with a reduction of the ability of the cells to adhere to the plate surface.

Seeking to investigate if miR-183 affects cell adhesion, not only in enforced in vitro experiments but also in unperturbed patient tissue, the expression of cell adhesion-annotated genes (Supplementary Table S5) and miR-183, as well as the status for different clinical parameters (biochemical recurrence, clinical M, clinical T, Gleason Score, pathologic N, pathologic T, PSA value, residual tumor and sample type) from normal, primary and metastatic tissue from PRAD-TCGA were analyzed by hierarchical clustering. The results show a predominantly negative association between the expression of the cell adhesion-annotated genes and the expression of miR-183, as well as with worse clinical parameters (represented in black) (Supplementary Figure S4 and Table S6), supporting an oncogenic negative modulation of cell adhesion by miR-183 in PrCa.

### 2.4. ITGB1 Regulation by MiR-183 May by Mediated by a Direct Interaction with the ITGB1 3′UTR in Prostate Cancer

**Figure 3:**
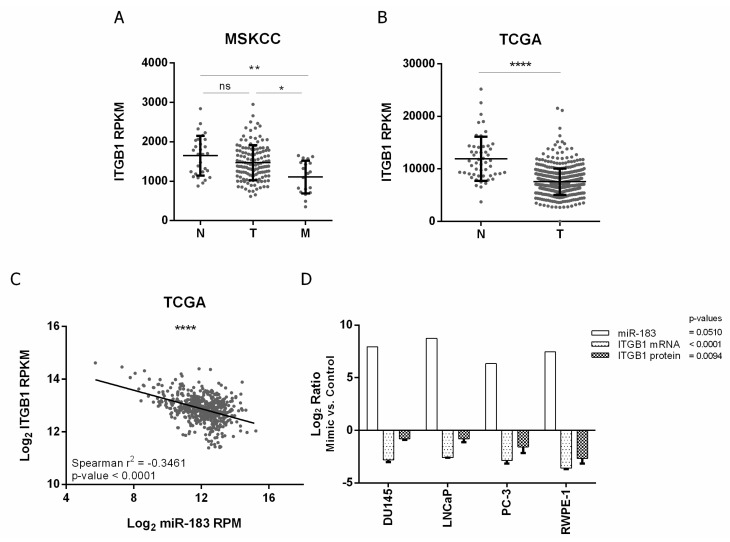
Expression of *ITGB1* and its association with miR-183 expression in PrCa. (**A**,**B**) association of *ITGB1* mRNA expression (RPKM) with tissue status in the MSKCC [28] and PRAD-TCGA cohorts, respectively. N = normal, T = primary tumor, M = metastatic tissue. A Mann–Whitney test was applied to assess the statistical significance of the difference between conditions. (**C**) Correlation between *ITGB1* mRNA (Log_2_ RPKM) and miR-183 (Log_2_ RPM) expression in metastatic, primary tumor and normal tissue samples from the PRAD-TCGA project, evaluated by a Spearman correlation test. (**D**) Effect of miR-183 on *ITGB1* expression in four PrCa cell lines. Cells were transfected with 20 nM of miR-183 mimic or control RNA. MiR-183 (normalized to RNAU6) and *ITGB1* mRNA (normalized to BACT and GAPDH) levels (2^−∆∆CT^) were assessed by RT-qPCR 72 h after transfection in 2 technical replicates per cell line. ITGB1 protein levels (mean fluorescence intensity) were assessed by flow cytometry 72 h after transfection in at least three independent experiments per cell line. The results of the ratio between miR-183 mimic and control are shown as Log_2_. A paired *t*-test was performed considering the four cell lines as biological replicates to assess the statistical significance between conditions. *p*-value * <0.05, ** <0.01, **** <0.0001, ns = not significant.

In order to study the molecular basis of the effect of miR-183 on cell adhesion, we selected the direct candidate target gene *ITGB1* for further studies, since it is one of the most overrepresented genes of the cell-adhesion-related enriched pathways (Table 2). In addition, the top enriched Wikipathway regulated by miR-183, EGF/EGFR, is known to crosstalk and share molecular components with the integrin signal [39]. Since the impact of *ITGB1* in cancer progression is controversial, we firstly studied the clinical relevance of *ITGB1* in PrCa. For that purpose, we analyzed the expression of *ITGB1* in metastatic, primary tumor and normal prostate patient samples from MSKCC and PRAD-TCGA cohorts. We found that *ITGB1* transcript was significantly underexpressed in metastatic tissue compared to tumor and normal tissue in MSKCC samples (*p*-value ≤ 0.05 and ≤0.01, respectively) and in tumor tissue compared to normal tissue in PRAD-TCGA samples (*p*-value ≤ 0.0001) (Figure 3A,B), suggesting a tumor suppressor role of *ITGB1* in PrCa. Furthermore, the expression of both miR-183 and *ITGB1* in the PRAD-TCGA samples is negatively correlated (Spearman r^2^ = −0.3461, *p*-value < 0.0001), in agreement with a repressive interaction between them.

To confirm the negative effect of miR-183 on *ITGB1* expression in vitro, *ITGB1*’s mRNA and protein levels were quantified after the forced overexpression of miR-183 in normal and tumor prostate cell lines relative to the control RNA. The mRNA level of *ITGB1* decreased 0.1 ± 0.0 (Log_2_ −2.8, 90%) in DU145, 0.2 ± 0.0 (Log_2_ −2.6, 80%) in LNCaP, 0.1 ± 0.0 (Log_2_ −2.9, 90%) in PC-3 and 0.1 ± 0.0 (Log_2_ 3.6, 90%) in RWPE-1 upon miR-183 mimic transfection (*p*-value < 0.0001). Accordingly, the protein level of ITGB1, measured by flow cytometry with an anti-ITGB1 antibody, was significantly reduced to 0.6 ± 0.0 (Log_2_ −0.8, 40%) in DU145, 0.6 ± 0.1 (Log_2_ −0.8, 40%) in LNCaP, 0.4 ± 0.1 (Log_2_ −1.6, 60%) in PC-3 and 0.2 ± 0.1 (Log_2_ −2.7, 80%) in RWPE-1 (*p*-value = 0.0094) (Figure 3D, Supplementary Figure S5). It is worth mentioning that we have attempted the inhibition of miR-183 levels with inhibitors (Dharmacon and Qiagen) at different concentrations and analytical times, but we have only observed a small increase of ITGB1 mRNA at 72 h post-transfection using a 200 μM concentration (Supplementary Figure S6A). Yet, when we analyzed ITGB1 protein by flow cytometry in the DU145 cell line, which was the one showing a higher upregulation of the mRNA level in our experiments, no modulation by miR-183 was observed (Supplementary Figure S6B).

**Figure 4:**
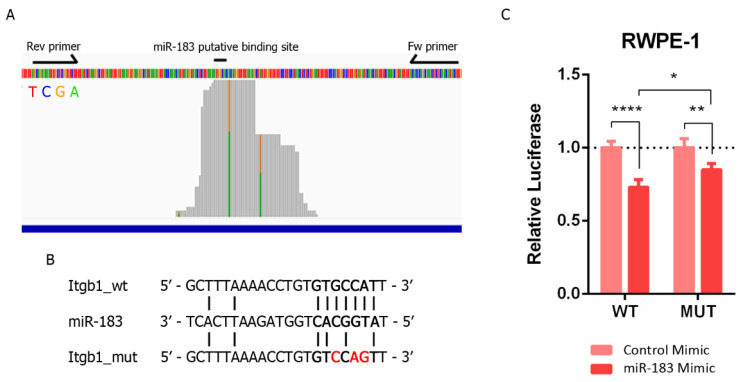
Validation of the direct miR-183 and *ITGB1* mRNA interaction in PrCa. (**A**) Mapping of prostate cell line reads along the putative binding site of miR-183 on *ITGB1*’s 3′UTR extracted from AGO-PAR-CLIP RNA-seq data [31]. The base identity is represented by four colors, as indicated. Perfect base matches and mismatches (due to base substitutions caused by the photoactivatable ribonucleoside crosslinking) are represented as gray and colored bars, respectively. Forward and reverse primers used to clone the miR-183 binding site of the *ITGB1* 3′UTR in pmiRGLO are indicated by arrows. The image is a modified screenshot of the IGV Software [42]. (**B**) Base pairing between miR-183 and the conserved miR-183 putative binding site on *ITGB1* wild type and mutated 3′UTR cloned in pmirGLO for reporter gene assays. (**C**) Reporter gene assay using pmiRGLO, bearing the region of the 3′UTR of *ITGB1* with the miR-183 wild type or mutated site, co-transfected with miR-183 mimic or control RNA in the RWPE-1 cell line. Luciferase activity was measured 48 h after transfection. The mean ± SD of four independent experiments is shown. A two-way ANOVA was applied to test the statistical significance of the differences between conditions. *p*-value * <0.05, ** <0.01, **** <0.0001.

We next sought to investigate if this repressive effect is due to a direct sequence-specific complementary interaction between *ITGB1* mRNA and miR-183 in the prostate tissue. We initially visualized the mapping distribution of AGO-PAR-CLIP reads containing the 6-mer miR-183 complementary site along the 3′UTR of the *ITGB1* gene from Hamilton et al. [31]. TargetscanHuman [40] predicts three sites for miR-183 in the 3′UTR of *ITGB1*: a 7merm8 site conserved among vertebrates (position 623–629 nt), an 8-mer site poorly conserved among vertebrates (position 1012–1019 nt) and a 6-mer site (position 83–87 nt). The most covered region of the *ITGB1* 3′ UTR bearing the 6-mer site complementary to the miR-183 seed in the AGO-PAR-CLIP dataset of PrCa cell lines turned out to be the 7merm8 binding site of miR-183 conserved among vertebrates (GUGCCAU) (Figure 4A, Supplementary Figure S7). The T-C transition identified in that AGO footprint occurs in 38% of the reads at the U base of *ITGB1* transcript, corresponding with position 9 of the miRNA (unpaired), consistent with the characteristics of the PAR-CLIP reads of prototypical miRNA interaction sites (which occur at U positions between the 8 and 13th 5′of the interaction site [41]). This mapping pattern supports a direct interaction of miR-183 on this *ITGB1* seed site in prostate cells. Thus, we cloned a 140 bp region of the *ITGB1* 3’UTR comprising this site into a luciferase reporter plasmid (Figure 4A) and introduced mutations in the 7merm8 conserved putative binding site for miR-183 (Figure 4B). The luciferase activity of the wild type (wt) *ITGB1* 3′UTR was significantly reduced to 0.73 when miR-183 was overexpressed by transfection in RWPE-1 cells compared to the transfection of a control RNA (*p*-value < 0.001). Furthermore, the mutation of three nucleotides in the putative binding site of miR-183 caused a partial loss of this repression to 0.85 (44% recovery, *p*-value < 0.05) (Figure 4C). 

Overall, the results indicate that a negative regulation of *ITGB1* by miR-183 may be operative in prostate cancer patient tissue, as well as in cell lines, and might contribute to prostate cancer biology. 

### 2.5. The Inhibition of Cell Adhesion Provoked by miR-183 May Be Due to a Reduction of Focal Adhesions Mediated by ITGB1 Downregulation

**Figure 5:**
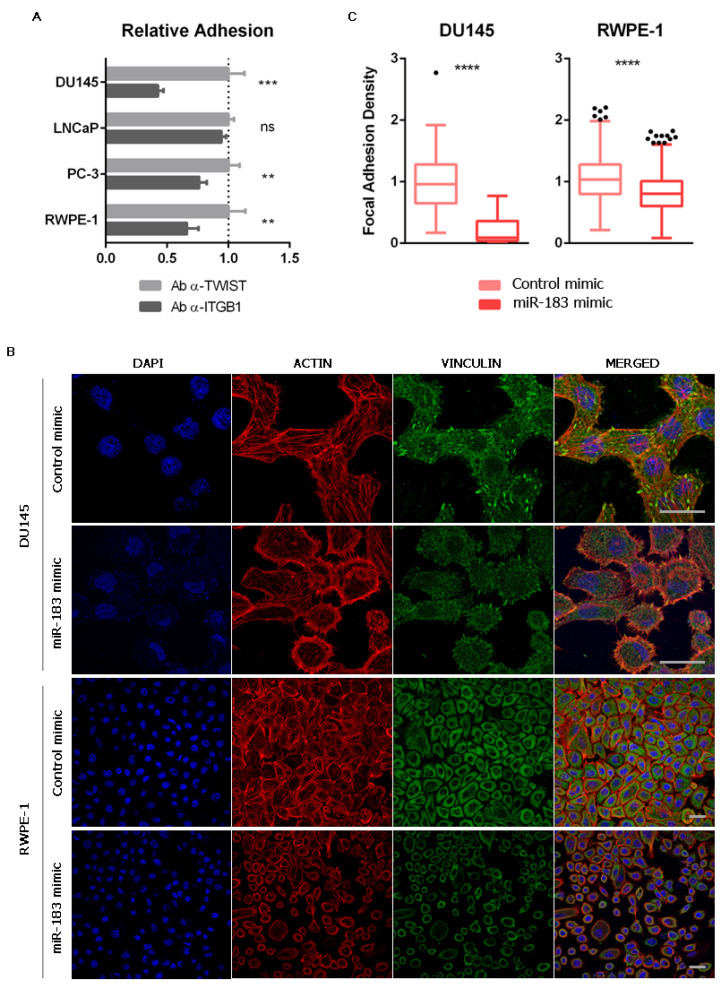
Effect of miR-183 on the number of focal adhesions. (**A**) Effect of ITGB1 blockade on cell adhesion. Untransfected cells were incubated with an antibody against ITGB1 (ab24693 Abcam) or TWIST (ab50887 Abcam), seeded on polystyrene 96-well plates and allowed to adhere for 0.5 or 1 h (depending on the cell line). Non-adherent cells were removed, and the remaining cells were quantified using CellTiter-Glo (Promega). The results of at least three independent replicates were normalized to the control and are shown as means ± SD. An unpaired t-test was applied to assess the statistical significance of the difference between conditions. (**B**) Immunocytochemistry of vinculin of prostate cells overexpressing miR-183. Seventy-two hours after transfection of 20 nM of miR-183, the cells were fixed, permeabilized and immunostained for vinculin using an Alexa Fluor 488-conjugated secondary antibody. Nuclei and filamentous actin were counterstained with DAPI and phalloidin-Alexa Fluor 568, respectively. The images were collected on a confocal microscope using a 63X oil immersion objective. Scale bars show 25 μm. Representative images are shown. (**C**) Effect of miR-183 on focal adhesion density. Quantification of focal adhesions per cell in vinculin immunocytochemical images of the DU145 and RWPE-1 cell lines. Images were processed using ImageJ. A Mann–Whitney test was applied to test the statistical significance of the difference between conditions. *p*-value ** <0.01, *** <0.001, **** <0.0001, ns = not significant.

Aiming to evaluate if the modulation of cell adhesion caused by miR-183 (Figure 2) is mediated by ITGB1, we first incubated untransfected prostate cells with a monoclonal antibody against ITGB1 known to block cell adhesion [43], or an antibody against the nuclear transcription factor TWIST as a control, and measured cell adhesion to the cell culture plate. A decrease in cell adhesion of 0.43 ± 0.05 (57%) in DU145, 0.76 ± 0.06 (24%) in PC-3 and 0.66 ± 0.09 (34%) in RWPE-1 was observed (Figure 5A), resembling the effect of miR-183 overexpression (Figure 2A). LNCaP cells did not change cell adhesion under these experimental conditions (Figure 5A). These results indicate that ITGB1 blockade produces a similar effect to miR-183 overexpression in this experimental setting, making it a suitable mediator of the miR-183 adhesive phenotype.

The interaction between the ECM and the integrins results in the assembly of specialized structures called focal adhesions (FA). They are highly specialized domains of the plasma membrane, consisting of clusters of integrins that form the closest contact with matrix proteins on the extracellular side, and represent the sites where converging actin filaments terminate and interact with integrins on the cytoplasmic side [44]. A variety of biological process involving changes in cell contractility require a change in the number and distribution of focal adhesions [45]. Interestingly, one of the most common integrins found in focal adhesions is α5β1 [46]. Considering that miR-183 and ITGB1 have opposite effects on cell adhesion and meet criteria for a miRNA/target RNA pair, we then investigated if miR-183 affects the number of the focal adhesions in the prostate cell line models. For this purpose, we measured FA using an antibody against the scaffold focal adhesion protein vinculin in DU145 and RWPE-1 cells overexpressing miR-183, employing identical conditions as those of the experiments shown in Figure 2. Indeed, we found a decrease in the number of FA per cell in cells overexpressing miR-183 relative to those overexpressing the control RNA (Figure 5B,C). This observation suggests that miR-183-mediated reduction of cell adhesion could be due to a reduction of the focal adhesions, which is consistent with a downregulation of *ITGB1*.

## 3. Discussion

Schaefer’s group, in 2009, was the first to identify miR-183 as deregulated in PrCa [15], and since then several miRNA expression profiling studies have unanimously proposed that the expression of miR-183 is positively associated with malignancy in PrCa [13,14,15,16,17,18,19,20,21,22,23,24,47,48]. We have also confirmed the oncogenic expression pattern of miR-183 in the largest PrCa cohorts publicly available (and in South American patients), and we found positive associations with clinical parameters that were not yet reported (PSA prior to radical prostatectomy, shorter time until recurrence, biochemical recurrence, clinical T stage and pathologic N status). Therein, we also found a positive association between miR-183 expression and PSA at diagnosis and biopsy, pathologic Gleason Score and pathological T stage, that has been reported before [13,14]. In agreement with these findings, an association between miR-183 expression with overall survival (OS) and disease free survival (DFS) was also demonstrated by Hua et al., in another PrCa cohort [20].

The current literature shows that miR-183 is involved in several processes relevant to PrCa progression, such as cell proliferation [14,20,25], migration [14,20,25], invasion [20,25], Zn metabolism [21,26] and docetaxel sensibility [19]. Yet, the relative contribution of miR-183 to these phenotypes and its relevance in PrCa has not been addressed. 

The goal of our study was to perform a de novo unbiased approach to discover the main pathways directly modulated by miR-183 in PrCa. We thus began the study with an integrated analysis of three independent datasets comprising: (1) genes responsible for miR-183 forced modulation in prostate cell lines, (2) transcripts containing the 6-mer (2–7 nt) sequence complementary to the miR-183 seed protected by AGO in prostate cell lines and (3) transcripts showing a significantly negative correlation with miR-183 expression in PrCa patient tissues. The intersection of the three gene lists resulted in 135 possible miR-183 direct target genes that turned out to be enriched in cell-adhesion-related terms, such as focal adhesion, proteoglycans, cell migration, mesenchymal–epithelial transition (MET) and regulation of the actin cytoskeleton. Cell-adhesion-related pathways, including focal adhesion, integrin signaling, tight junction and regulation of the actin cytoskeleton have been associated with prostate tumorigenesis and, interestingly, miR-183 has been one of the top miRs predicted to be involved in these pathways by the theoretical network analysis of PrCa [13,49,50]. Coincidentally, Dambal et al., found that cell adhesion is the main pathway downregulated in the RWPE-1 cell line overexpressing the full miR-183 cluster (miR-96, miR-182 and miR-183 simultaneously) [26]. Yet, they observed a slight upregulation of cell adhesion in vitro in RWPE-1 while observing a statistically non-significant decrease in cell adhesion in RWPE-2 and PC3 cells, suggesting that the cell adhesion assay used is not sensitive to the cell adhesion phenotype predicted from the gene enrichment study. In addition, the effect of miR-183 as a promoter of cell proliferation, migration and invasion that was reported previously in PrCa [14,20,25] could be reliant on the changes in cell adhesion we are describing here. Through the overexpression of miR-183 in prostate cell lines followed by attachment and spreading assays, we have validated that miR-183 negatively modulates cell adhesion in vitro. Furthermore, the association between miR-183 and cell-adhesion-related gene expression in patient samples strongly suggests that these in vitro observations could be also occurring in the clinical set.

Aiming to study the molecular basis of the effect of miR-183 on cell adhesion, we selected the direct candidate target gene *ITGB1* for further studies, since it is the most overrepresented gene among the cell-adhesion-related pathways enriched in the 135 genes modulated by miR-183. Interestingly, of the four direct targets of miR-183 already reported in PrCa (*DKK3*, *SMAD4*, *TPM1* and *SPRY2*, listed in Supplementary Table S7) only *TPM1* satisfies the three criteria used to select direct target genes in our study, although its transcript is only modulated in the DU145 cell line transfected with the miR-183 inhibitor. Nevertheless, *DKK3*, *SMAD4* and *SPRY2* met only two direct target criteria in our study. Although methodological differences may explain the discrepancies with our findings, it is also possible that these genes are bona fide but indirect targets of miR-183 repression. Therefore, *ITGB1* has more support as a direct target of miR-183 repression using our selection pipeline than the previously reported direct target genes.

Although the validation of predicted direct microRNA targets is commonly performed by a reporter gene assay for single target identification, AGO2 pull down or biotinylated-microRNA pull down followed by RT-qPCR of both RNAs have been shown to provide additional support. Thus, a limitation of the studies that identified miR-183 targets in PrCa (including ours), as well as those that proposed the direct regulation of ITGB1 by miR-183 in other tissues, is the reliance in a sole reporter gene assay to discriminate the direct vs. the indirect regulation exerted by miR-183.

ITGB1 is a member of the integrin family, which is composed of the major cell surface receptors that mediate adhesion to the ECM. Integrins are expressed in all nucleated cells of the human body and are involved in key biological processes, such as cell differentiation, cell adhesion, cell migration, cell proliferation and cell survival, thus playing fundamental roles in cancer [51]. Although the role of integrins in cancer progression is controversial and likely tissue- and time-specific [51,52], in this study we show that *ITGB1* has an expression pattern of a tumor suppressor gene in prostate cancer tissues. Furthermore, we found a significant correlation between miR-183 and *ITGB1* expression in the PRAD-TCGA samples with a Spearman correlation coefficient of -0.3, similar to that reported for already validated miRNA-target pairs in PrCa [53]. Indeed, we demonstrated that miR-183 modulates *ITGB1* expression at both the mRNA and protein levels in vitro. Furthermore, the observation of AGO2 footprints protecting the conserved 7merm8 site for miR-183 in the 3′UTR of *ITGB1*, together with reporter gene assays showing the dependence of the reporter activity on this site to respond to miR-183, indicate that the *ITGB1* transcript may be a direct target of repression by miR-183 in prostate cells. Additionally, we demonstrated that the antibody-mediated blocking of ITGB1 decreases the adhesion of DU145, PC-3 and RWPE-1 cells, in a similar mode as the overexpression of miR-183, suggesting that ITGB1 could mediate the effect on cell adhesion produced by the overexpression of miR-183 in these cell lines, whereas in LNCaP it may be mediated by a mechanism independent of ITGB1. Interestingly, the LNCaP cell line has lower expression of ITGB1 than DU145 and PC-3 cell lines, according to the data from MSKCC [28] and the Expression Atlas database [54], thus it might be less dependent on ITGB1 for adhesion to the plate surface. Alternatively, miR-183 may be affecting cell adhesion at different levels, through additional direct targets of repression and indirectly regulated genes. A closer look into the mechanism of cell adhesion mediated by ITGB1 demonstrated that the overexpression of miR-183 reduces the number of focal adhesions per cell, providing additional evidence of ITGB1-mediated regulation of the focal adhesion pathway in response to miR-183. 

*ITGB1* has been proposed as a direct target of repression by miR-183 in cervical [55] and endometrial cancer [56], but in both tissues miR-183 acts as a tumor suppressor causing upregulation of the invasiveness promoter ITGB1. Likewise, Sun et al., proposed that the oncogenic lncRNA *MALAT1* functions as a sponge of tumor suppressor miR-183, leading to the overexpression of *ITGB1* by the reduction of direct targeting in melanoma [57]. A similar regulation was proposed for the circular RNA hsa_circ_0000291 and the hsa_circ_0000291-miR-183-*ITGB1* axis in gastric cancer, affecting proliferation and cell migration in vitro [58]. Thus, the available evidence of the direct targeting of *ITGB1* by miR-183 was achieved in a scenario with the tumor suppressor miR-183 and oncogenic *ITGB1*.

Although miR-183 has a well proven oncogenic function in PrCa, the tumor suppressor role of *ITGB1* suggested by our results is contradictory with previous findings. Indeed, *ITGB1* knockdown by RNAi in PrCa cell lines inhibits migration, invasion [59] and EMT [60], and sensitizes cells to ionizing radiation [61]. Methodological differences in the *ITGB1* silencing (RNAi, miR-183 and antibody blockage), leading to different molecular/cellular effects, may be responsible for the conflicting results. Since it has been proposed that integrins could have a dual role during carcinogenesis, functioning as oncogenics or as tumor suppressors, depending on the microenvironment [52], perhaps the experimental conditions used in the different experiments mimic different cell contexts in a yet unknown way. Likewise, *ITGB1* has been shown to be a tumor suppressor in breast cancer and PrCa when associated with α2 subunits forming the heterodimeric α2β1 integrin, a receptor for collagen and other matrix molecules [62], regulating the early steps of metastasis. Another study on PrCa reported that integrin α3β1 inhibited cancer cell metastasis by regulating the Hippo signaling pathway [63]. Nevertheless, although further studies are needed to understand these discrepancies, the expression of *ITGB1* and miR-183 and their association to disease status in our study and previous miRNA studies strongly favor a tumor suppressor for ITGB1 and oncogenic role for miR-183 in PrCa.

## 4. Materials and Methods

### 4.1. Human Specimens

Tissue sections from seven radical prostatectomies were obtained from the Department of Anatomic Pathology of the Police Hospital, Montevideo, Uruguay. This study was approved by the Institutional Review Board of the Hospital Policial, D.N.AA.SS., Montevideo, Uruguay (2010). Patient consent was waived because the samples were archived prostatectomies that were already available at the Anatomic-Pathology Department of the Hospital as part of a collection, so the pathological specimens existed prior to the current study and no patient data was revised. The identity of the subjects was not disclosed. Paraffin fixed blocks stained with hematoxylin and eosin (H&E) were evaluated by two independent pathologists. The samples were selected by histopathological criteria, consisting in the presence of clearly discernible normal and tumor regions that do not present areas of heterogeneous tissue composition (hyperplasia, parenchyma, or vascularization). Unstained sections of 10-μm thickness, contiguous to the sections selected by the pathologist, were then freshly obtained to extract small RNAs using the RNeasy FFPE (Qiagen) Kit, with the following modifications: two extra washes with xylene and absolute ethanol were added. The RNA was resuspended in RNase-free water and stored at −20 °C for further analysis.

### 4.2. Cell Lines

DU145, LNCaP (ATCC CRL-1740), PC-3 and RWPE-1 human prostate cancer cell lines were obtained from ATCC (Manassas, VA, USA). LNCaP, DU145 and PC-3 cells were maintained in Roswell Park Memorial Institute Medium 1640 with stable glutamine (Capricorn SC.) supplemented with 10% fetal bovine serum (Capricorn SC.) and penicillin/streptomycin. The RWPE-1 cell line was cultured in keratinocyte serum-free medium (Gibco by Life Technologies, Carlsbad, CA, USA) supplemented with 0.03 mg/mL of bovine pituitary extract (BPE) and 0.5 ng/mL of EGF human recombinant epidermal growth factor (EGF) and penicillin/streptomycin. All cell lines were maintained in a 5% carbon dioxide atmosphere at 37 °C.

### 4.3. Cell Ttransfection

#### 4.3.1. Microarrays Experiments

50 nM of miScript hsa-miR-183-5p mimic, 50 nM Allstar Negative Control siRNA and 500 nM miScript hsa-miR-183-5p inhibitor (Qiagen, Germantown, MD, USA) were transfected into the LNCaP and DU145 cells using FuGENE (Roche, Middleton, WI, USA), following the manufacturer’s instructions.

#### 4.3.2. Functional Analysis

20 nM MiRIDIAN hsa-miR-183-5p mimic or miRNA Negative Control (CN-001000-01-05) (Dharmacon, Lafayette, LA, USA) were transfected into DU145, LNCaP, PC-3 and RWPE-1 using Lipofectamine 3000 (Invitrogen, Waltham, MA, USA), following the manufacturer’s instructions.

200 nM miRIDIAN miR-183 Hairpin Inhibitor (Dharmacon), miRCURY LNA miRNA miR-183 Power Inhibitor (Qiagen) and the respective controls were transfected into DU145, LNCaP, PC-3 and RWPE-1 using Lipofectamine 3000 (Invitrogen), following the manufacturer’s instructions.

#### 4.3.3. Reporter Gene Assays

5 nM MiRIDIAN hsa-miR-183-5p mimic or miRNA Negative Control (CN-001000-01-05) (Dharmacon) and 125 ng of pmiRGLO-3′UTR-*ITGB1*-wt or -mut were transfected into RWPE-1 cells using Lipofectamine 3000 (Invitrogen) following the manufacturer’s instructions. 

### 4.4. Gene Expression Microarrays

Total RNA was extracted 24 h after transfection with TRIzol, following the manufacturer’s instructions. The integrity of the RNA samples was evaluated by electropherogram analysis in a 2100 Bioanalyzer with Agilent 2100 Expert software (Agilent Technologies, Santa Clara, CA, USA). The RNA was hybridized onto the Affymetrix Gene Chip (HG-U133A 2.0). Quality control analysis and pre-processing of the .cel files microarray expression data was performed in Chipster [64], following the manufacturer’s guidelines. Normalization was performed using the RMA algorithm and annotation using the general Affymetrix probe set library in Chipster [64]. Normalized Log_2_ expression values were used to calculate the fold change related to Allstar Negative Control siRNA-transfected cells. Transcripts showing a FC ≥ 1.25 in at least two out of the four comparisons (miScript hsa-miR-183-5p Mimic and miScript hsa-miR-183-5p inhibitor related to Allstar Negative Control siRNA in DU145 and LNCaP cells) were selected for further analysis (Supplementary Table S1).

### 4.5. Dataset Analysis

Gene and miRNA microarray expression data, as well as the status for different clinical parameters from the MSKCC project [28] (14 metastatic, 99 primary tumor and 28 normal adjacent benign prostate samples), were downloaded from the Gene Expression Omnibus (GEO) repository (accession number: GSE21036) [65]. 

The AGO-PAR-CLIP sequencing data of human prostate cell lines (DU145, LNCaP, PC-3, 22RVI and LAPC4) from Hamilton et al. [31] were downloaded from the Sequence Read Archive (SRA) [66] (accession numbers: DU145: SRR3502975; LNCaP: SRR3502923, SRR3502926, SRR3502931; PC-3: SRR3502951, SRR3502954; 22RVI: SRR3502967, SRR3502969, SRR3502970; LAPC4: SRR3502955, SRR3502965). Adapter removal, quality control and trimming were performed using miARma software [67] with default parameters. Reads between 12 and 50 bp containing the 6-mer (2–7 nt) sequence complementary to the miR-183 seed (5′-TGCCAT-3′) were selected using a custom Bash script and aligned to human genome GRCh38.p13 using Bowtie2 [68], allowing two mismatches (since T-C transitions are caused by crosslinking in PAR-CLIP experiments, and their frequency is approximately two per footprint). Total read counts for each gene transcript were obtained with HT-seq Count [69] using default parameters. Gene transcripts with at least one miR-183 site read in at least three out of the five analyzed cell lines were selected (Supplementary Table S2). The AGO2 protected regions of the *ITGB1* genes were also visualized in IGV Software [42].

Normalized RNA-seq data of miR-183 and mRNA (19076 transcripts) expression, as well as the status for different clinical parameters from the PRAD-TCGA cohort (1 metastatic, 490 primary tumor and 52 normal prostate patient samples), were downloaded from the Xena Browser [70]. For the massive analysis of the PRAD-TCGA samples, raw RNA seq data was downloaded from the FireBrowse repository (http://firebrowse.org/, accessed on 21 April 2020) and the Spearman correlation and *p*-values were calculated using the Scipy library [71] in Python. Transcripts with null reads were excluded for the calculations. Transcripts showing a negative correlation with miR-183 (r^2^ < 0) and a *p*-value ≤ 0.05 were selected for further analysis (Supplementary Table S3).

The list of cell-adhesion-annotated genes was downloaded from the AmiGO 2 repository [72] (http://amigo.geneontology.org/, accessed on 23 April 2020) (Supplementary Table S5). Normalized RNA-seq data of the cell-adhesion-annotated genes (RPKM) and miR-183 (RPM), as well as the status for different clinical parameters (biochemical recurrence, clinical M, clinical T, Gleason score, pathologic N, pathologic T, PSA value, residual tumor, and sample type) from 1 metastatic, 490 primary tumor and 52 normal prostate patient samples generated by the TCGA project (http://cancergenome.nih.gov/, accessed on 23 April 2020), was downloaded from the Xena Browser [70] (Supplementary Table S6). A heatmap of hierarchical clusterization was performed using Euclidean distance and a Spearman rank correlation algorithm for rows and columns, respectively, using Morpheus (https://software.broadinstitute.org/morpheus, accessed on 23 April 2020).

### 4.6. Biological Term Enrichment Analysis of the Direct Candidate Targets Genes of MiR-183 

The list of 135 direct candidate target genes were analyzed for enrichment of Wikipathways, Kyoto Encyclopedia of Genes and Genomes (KEGG) pathways and GO Cellular Components using the online analysis tool Enrichr [37,38]. Adjusted *p*-values were used for filtering the top 10 enriched terms.

### 4.7. Cell Adhesion Assays

#### 4.7.1. Quantitative Assessment

After 72 h of transfection, the cells were harvested by trypsinization and 2E4 cells per well were seeded on polystyrene 96-well plates (Greiner CELLSTAR Cat. No. 655 180, Kremsmünster, Austria), which have a physical surface treatment for improving cell attachment, and were allowed to adhere for 0.5 or 1 h (depending on the cell line) in a 5% carbon dioxide atmosphere at 37 °C. After the adhesion time, non-adherent cells were removed by washing with pre-warmed fresh media and initial and remaining (attached) cells were quantified in independent wells using CellTiter-Glo (Promega, Madison, WI, USA), following the manufacturer’s instructions, in Varioskan Flash equipment (Thermo Electron Corporation, Waltham, MA, USA). Three independent experiments were performed for each cell line. The fraction of attached cells in miR-183 mimic-transfected cells was normalized to control for each cell line. 

#### 4.7.2. Qualitative Assessment

After 72 h of transfection, the cells were harvested by trypsinization and 2E4 cells per well were seeded on coverslips and allowed to adhere for 3 h in a 5% carbon dioxide atmosphere at 37 °C. After the adhesion time, the cells were fixed with cold 4% paraformaldehyde (PFA) for 15 min at room temperature (RT) and blocked and permeabilized with 3% BSA, 2% Glycine and 0.3% Triton X-100 in PBS for 1 h at 37 °C. After that, the cells were incubated with an Alexa Fluor 488-conjugated phalloidin (1/400, Invitrogen) for 30 min at RT for actin cytoskeleton visualization and mounted in a 90% glycerol Tris HCl 0.05 M pH = 8 medium. Images of six representative fields for each condition were captured in a ZOE Fluorescent Cell Imager (Biorad) with a monochrome camera of 12-bit CMOS 5 megapixels with a magnification of 175× and processed with Fiji Software [73] for area and circularity calculations. At least 300 cells were analyzed for each cell line in 1 experiment. 

### 4.8. RNA Extraction, Reverse Transcription and Quantitative Real-Time PCR

Total RNA from cell lines was extracted 24 h after transfection using the miRNAeasy kit (Qiagen), following the manufacturer’s instructions. 

For miR-183 quantification, 1 μg of RNA was used for reverse transcription with the miScript II RT kit (Qiagen). Quantitative real-time PCR (qRT-PCR) was performed using the QuantiTect SYBR Green PCR kit (Qiagen) and miScript Primer Assays (Qiagen). RNAU6 or SCARNA17 were used as the internal controls of RNA load.

For mRNA quantification, 200 ng of total RNA was used for reverse transcription with SuperScript III (Invitrogen), using 100 ng of random primers. qRT-PCR was performed using SensiFast SYBR Hi-Rox (Bioline, London, UK) and specific gene primer pairs: Itgb1 forward primer 5′-CGATGCCATCATGCAAGT-3′, Itgb1 reverse primer 5′-ACACCAGCAGCCGTGTAAC-3′, Gapdh forward primer 5′-CCCCGGTTTCTATAAATTGAGC-3′, Gapdh reverse primer 5′-CACCTTCCCCATGGTGTCT-3′, β-Actin forward primer 5′-CCAACCGCGGAGAAGATGA-3′ and β-Actin reverse primer 5′-CCAGAGGCGTACAGGGATAG-3′. Gapdh and β-Actin were used as the internal controls of RNA load. 

The relative quantification was attained using the 2^−ΔΔCt^ method [74], in StepOne Real-Time PCR System equipment (Applied Biosystems, Waltham, MA, USA).

### 4.9. Flow Cytometry for ITGB1 Quantification

After 72 h of transfection, the cells were harvested by trypsinization and incubated with an anti-ITGB1 monoclonal antibody (1/200, ab24693 Abcam, Cambridge, UK) in 0.2% BSA in PBS for 30 min at 4 °C with gentle shaking. Next, the cells were washed and then incubated with anti-mouse Alexa Fluor 488 (1/1000, A-11029 Invitrogen) for 30 min at 4 °C with gentle shaking. Isotype control using untransfected cells was performed for each cell line. Next, the cells were washed and resuspended in 1% BSA 0.5 mM EDTA in PBS. A total of 10,000 events per sample were acquired in an Accuri™ C6 flow cytometer (BD Bioscience, Franklin Lakes, NJ, USA). The mean fluorescence intensity in the FL1 channel (488 nm laser and 515 ±15 nm emission filter) was used as the ITGB1 protein level. Three independent experiments were performed for each cell line. The results are shown as the ratio of ITGB1 protein level between the miR-183 mimic and the control transfected cells.

### 4.10. Luciferase Reporter Gene Assay

Reporter plasmids bearing the 3′UTR of wild type and mutated *ITGB1* were constructed as follows. The 3′UTR of *ITGB1* was amplified from human genomic DNA using the following primers: MssI-NotI-3′UTR-*ITGB1* forward primer 5′-GTTTAAACGCGGCCGCAGTATGTTGAGAGTTGCTGGTGT-3′ and XbaI-3′UTR-*ITGB1* reverse primer 5′-TCTAGAAGCAGAAAATTGCTCGGTTCT-3′. Mutations in the putative binding site of miR-183 were introduced by Seed Mutagenesis Assembly PCR (SMAP), as described in [75], using the partially overlapping primers complementary to the putative binding site of miR-183: *ITGB1*mut forward primer 5′-GCTTTAAAACCTGTGTCCAGTTTTAAGAGTTACTTAATG-3′ and *ITGB1*mut reverse primer 5′-CATTAAGTAACTCTTAAAACTGGACACAGGTTTTAAAGC-3′. Cloning of the 3′UTR wild type and mutated sequences into pmiRGLO Dual-Luciferase miRNA Target Expression Vector (Promega, Madison, WI, USA) was performed by MssI and XbaI digestion. NotI digestion was used for colony screening. The sequences of the constructed plasmids were verified by Sanger Sequencing (Macrogen, Seoul, South Korea). Luciferase Assay was performed 48 h after transfection with Dual-Luciferase Reporter Assay System (Promega) following the manufacturer’s instructions and the luminescence was measured with Varioskan Flash equipment (Thermo Electron Corporation, Waltham, MA, USA) using the default parameters. Four independent experiments were performed. Results are shown as the ratio between Firefly and Renilla luminescence of the miR-183 mimic relative to the control transfected cells.

### 4.11. ITGB1 Blockade

Untransfected DU145, LNCaP, PC-3 and RWPE-1 cells were harvested by trypsinization and 4 × 10^5^ of each were incubated with anti-ITGB1 (5 μg/mL, ab24693 Abcam) or anti-TWIST (5 μg/mL, ab50887 Abcam) in 0.2% BSA in PBS for 30 min at 37 °C with gentle shaking. Next, the cells were washed 3 times with PBS 1 × and 2 × 10^4^ cells were seeded per well on polystyrene 96-well plates for adhesion. Then, the quantitative cell adhesion assay was performed, as described before.

### 4.12. Focal Adhesion Quantification

After 72 h of transfection, the cells were fixed with 4% PFA for 15 min at RT and permeabilized in 3% BSA, 2% Glycine and 0.3% Triton X-100 for 1 h at 37 °C. Next, the cells were incubated with anti-vinculin (1:100, Ab18058 Abcam) for 1 h at 37 °C. After washing three times, the cells were incubated in anti-mouse Alexa Fluor 488 (1/1000, A-11029 Invitrogen) with normal goat serum (1:20, Invitrogen). After washing three times, actin and nuclei were counterstained with phalloidin-Alexa Fluor 568 for 30 min at RT (1/400, Invitrogen) and DAPI for 15 min at RT. The preparations were then mounted with ProLong Diamond Antifade mounting medium (Thermo Scientific). Images were acquired with a ZEISS (LSM 800 AiryScan) confocal microscope. All images were then processed automatically in batches with Fiji Software [73]. The vinculin signal was used to quantify the number of focal adhesions per cell using background subtraction and applying color and size thresholds, whereas actin and nuclei staining were used to identify the individual cells. Three independent experiments were performed for each cell line. The results of at least 50 cells from one representative experiment are shown in a box plot using the Tukey method.

### 4.13. Statistical Analysis

A D’Agostino–Pearson test was conducted to assess the distribution of the data. The statistical analysis chosen for each experiment is referred to on each figure. All the analyses were performed in GraphPad Prism 6. The observed differences were expressed using *p*-values. Results with a *p*-value < 0.05 were considered significant (* *p* < 0.05, ** *p* < 0.01, *** *p* < 0.001, **** *p*-value < 0.0001).

## 5. Conclusions

Our study demonstrated that miR-183 modulates the adhesion of prostate cells, reducing the number of focal adhesions and altering the structure of the actin cytoskeleton. Furthermore, it provides evidence supporting *ITGB1* as a direct target of repression by miR-183 in prostate cells and proposes that their interaction contributes to the reduction of cell adhesion caused by the upregulation of the miR-183.

## Figures and Tables

**Table 1:** Expression of miR-183 in the MSKCC [28] and PRAD-TCGA cohorts and its association with clinical variables. The Mann–Whitney test was applied to assess the statistical significance of the difference between conditions.

Cohort	Clinical Parameter	Conditions Compared	Ratio between Conditions	*p*-Value
MSKCC	Tissue type	Primary tumor vs. Normal	3.3 ± 2.4	<0.0001
		Metastasis vs. Normal	5.3 ± 4.1	<0.0001
	PSA at diagnosis	High vs. Low	1.5 ± 1.1	0.0182
	Preoperative PSA	High vs. Low	2.5 ± 1.9	<0.0001
	Time until recurrence (month)	Shorter vs. Longer	1.3 ± 1.9	0.0195
PRAD-TCGA	Tissue type	Primary tumor vs. Normal	4.3 ± 3.0	<0.0001
	Biochemical recurrence	YES vs. NO	1.3 ± 1.1	0.0404
	Clinical T	T3-T4 vs. T1-T2	1.5 ± 1.3	0.0020
	Gleason Score	8-9-10 vs. 6-7	1.4 ± 1.0	<0.0001
	Pathologic N	N1 vs. N0	1.3 ± 0.9	0.0030

**Table 2:** Biological term enrichment analysis of the 135 miR-183 candidate direct target genes.

Wikipathways		
Term	Adjusted *p*-Value	Genes
EGF/EGFR Signaling Pathway WP437	3.59 × 10^−5^	*MAP3K2;MAPK7;GRB2;PTPN11;IQGAP1;CBL;CRK;SOS2;AP2M1;EGFR*
Focal Adhesion WP306	4.92 × 10^−5^	*ITGB1;CCND1;PIK3CA;CTNNB1;GRB2;ARHGAP5;CRK;ACTB;RHOA;EGFR;VEGFA*
RAC1/PAK1/p38/MMP2 Pathway WP3303	5.91 × 10^−5^	*ITGB1;PIK3CA;CTNNB1;PTPN11;GRB2;CRK;EGFR*
VEGFA-VEGFR2 Signaling Pathway WP3888	7.15 × 10^−5^	*ITGB1;CCND1;PIK3CA;CTNNB1;GRB2;PTPN11;IQGAP1;CBL;CRK;RHOA;VEGFA*
Regulation of Actin Cytoskeleton WP51	7.80 × 10^−5^	*PIK3CA;RDX;IQGAP1;CRK;SOS2;ACTB;RHOA;EGFR;SSH1*
Signaling of Hepatocyte Growth Factor Receptor WP313	1.82 × 10^−4^	*ITGB1;PIK3CA;PTPN11;GRB2;CRK*
MET in type 1 papillary renal cell carcinoma WP4205	1.92 × 10^−4^	*PIK3CA;PTPN11;GRB2;CBL;SOS2;CRK*
ErbB Signaling Pathway WP673	2.17 × 10^−4^	*CCND1;PIK3CA;GRB2;CBL;SOS2;CRK;EGFR*
Endometrial cancer WP4155	2.20 × 10^−4^	*CCND1;PIK3CA;CTNNB1;GRB2;SOS2;EGFR*
ESC Pluripotency Pathways WP3931	5.93 × 10^−4^	*MAPK7;LRP5;CTNNB1;GRB2;PTPN11;IL6ST;EGFR*
**KEGG**		
Proteoglycans in cancer	2.75 × 10^−8^	*ITGB1;RDX;PTPN11;IQGAP1;CBL;ACTB;RHOA;EGFR;VEGFA;CCND1;PIK3CA;CTNNB1;GRB2;SOS2P*
Focal adhesion	1.65 × 10^−6^	*ITGB1;CCND1;PIK3CA;CTNNB1;GRB2;ARHGAP5;CRK;SOS2;ACTB;RHOA;EGFR;VEGFA*
Human cytomegalovirus infection	4.31 × 10^−6^	*AKAP13;CCND1;PIK3CA;GNA11;CREB3L2;CTNNB1;GRB2;CRK;SOS2;RHOA;EGFR;VEGFA*
Chronic myeloid leukemia	4.99 × 10^−5^	*CCND1;PIK3CA;PTPN11;GRB2;CBL;SOS2;CRK*
Bacterial invasion of epithelial cells	5.19 × 10^−5^	*ITGB1;PIK3CA;CTNNB1;CBL;CRK;ACTB;RHOA*
Regulation of actin cytoskeleton	8.63 × 10^−5^	*ITGB1;PIK3CA;RDX;IQGAP1;CRK;SOS2;ACTB;RHOA;EGFR;SSH1*
Colorectal cancer	9.66 × 10^−5^	*CCND1;PIK3CA;CTNNB1;GRB2;SOS2;RHOA;EGFR*
Endometrial cancer	9.88 × 10^−5^	*CCND1;PIK3CA;CTNNB1;GRB2;SOS2;EGFR*
Prostate cancer	1.45 × 10^−4^	*CCND1;PIK3CA;CREB3L2;CTNNB1;GRB2;SOS2;EGFR*
Renal cell carcinoma	2.20 × 10^−4^	*PIK3CA;PTPN11;GRB2;SOS2;CRK;VEGFA*
**GO Cellular Component**		
Focal adhesion (GO:0005925)	6.05 × 10^−5^	*NUP214;ITGB1;RDX;ADAM10;IQGAP1;LPP;ACTB;RHOA;EGFR;SENP1;MPRIP;ANXA6;CTNNB1;SNTB2*

## Data Availability

Gene and miRNA microarray expression data, as well as the status for different clinical parameters from the MSKCC project [28], were downloaded from the Gene Expression Omnibus (GEO) repository (accession number: GSE21036) [65]. The AGO-PAR-CLIP sequencing data of human prostate cell lines (DU145, LNCaP, PC-3, 22RVI and LAPC4) from Hamilton et al. [31] were downloaded from the Sequence Read Archive (SRA) [66] (accession numbers: DU145: SRR3502975; LNCaP: SRR3502923, SRR3502926, SRR3502931; PC-3: SRR3502951, SRR3502954; 22RVI: SRR3502967, SRR3502969, SRR3502970; LAPC4: SRR3502955, SRR3502965). Normalized RNA-seq data of miR-183 and mRNA expression, as well as the status for different clinical parameters from the PRAD-TCGA cohort, were downloaded from the Xena browser (https://xenabrowser.net/, accessed on 21 April 2020). The list of cell-adhesion-annotated genes was downloaded from the AmiGO 2 repository [72] (http://amigo.geneontology.org/, accessed on 23 April 2020).

## Supplementary Materials

The following are available online at https://www.mdpi.com/article/10.3390/ncrna8010011/s1, Figure S1: Analysis of miR-183 expression between primary tumor and normal adjacent tissue and association with Gleason score in radical prostatectomies from seven Uruguayan patients. Figure S2: Association between miR-183 expression and clinical variables in the MSKCC [28] and PRAD-TCGA cohorts. Figure S3: miR-183 expression in the samples used for gene expression microarray experiments. Figure S4: Hierarchical clustering of the PRAD-TCGA samples, based on the expression of miR-183 and cell adhesion genes, as well as the status of different clinical parameters. Figure S5: Flow cytometry analysis of DU145 cells for the quantification of ITGB1 protein. Figure S6: Regulation of ITGB1 expression by miR-183 inhibitors. Figure S7: Mapping of the prostate cell line AGO-PAR-CLIP reads extracted from Hamilton et al. Table S1: List of mRNA transcripts repressed by miR-183, identified by gene expression Affymetrix microarrays. Table S2: List of mRNA transcripts immunoprecipitated with the AGO2 antibody that map onto conserved canonical miR-183 seeds (6 mer and longer) in prostate cell lines. Table S3: List of mRNA transcripts negatively correlated with miR-183 in the PRAD-TCGA samples. Table S4: List of the 135 direct candidate target genes of miR-183, resulting from the intersection of selected genes of Tables S1–S3. Table S5: List of cell-adhesion-annotated genes from the AMIGO2 GO repository. Table S6: Matrix of normalized RNA-seq data of the cell-adhesion-annotated genes (RPKM) and miR-183 (RPM) as well as the status for different clinical parameters from the PRAD-TCGA project used for hierarchical clusterization. Table S7: List of miR-183-directed targets published in PrCa indicating their compliance to the criteria used to select direct targets in our study.
