# Supplementary material for: Hsa-miR-183-5p Modulates Cell Adhesion by Repression of ITGB1 Expression in Prostate Cancer
Source: Noncoding RNA. 2022 Jan 18;8(1):11. doi: 10.3390/ncrna8010011 (PMC8875343; doi:10.3390/ncrna8010011)
Supplement: Supplementary file 1 [file ncrna-08-00011-s001.zip › Oliveira-Rizzo et al ncRNA Supplementary Figures and Table captions_CO.pdf]

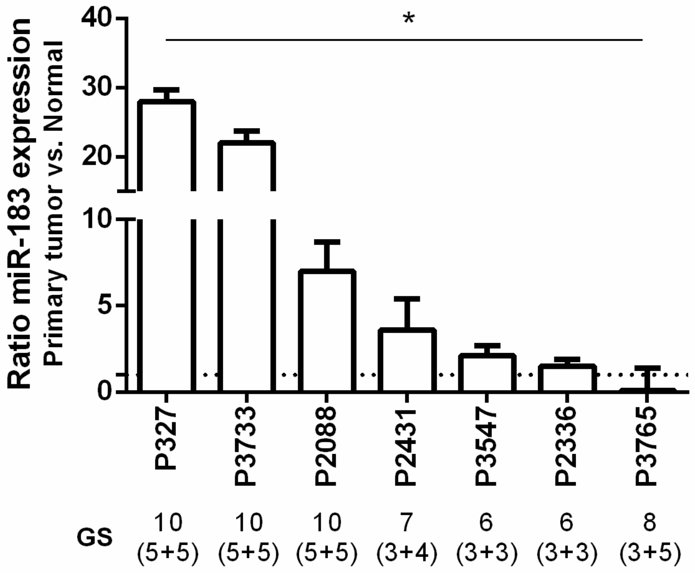

Supplementary Figure S1. Analysis of miR-183 expression between primary tumor and normal adjacent tissue and association with Gleason Score in radical prostatectomies from 7 Uruguayan patients. MiR-183 expression level was assessed by RT-qPCR, using SCARNA17 RNA for normalization and the difference between matched normal and tumor tissue are shown ($2^{-\Delta\Delta CT}$). Wilcoxon matched pairs signed rank test was applied to assess the statistical significance of the difference between conditions.

A

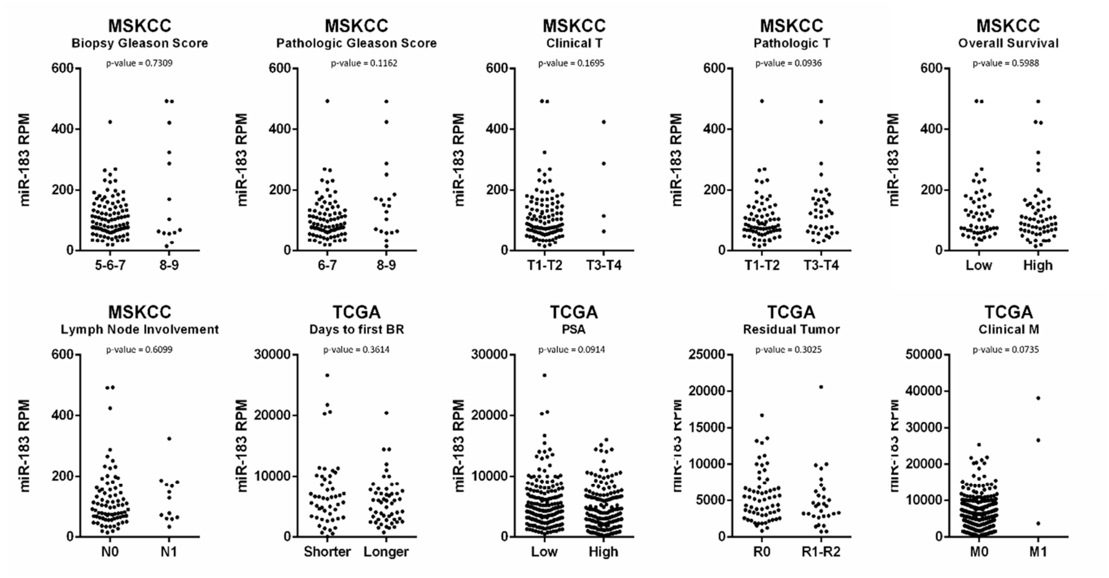

B

miRNA Name	Cancer Abbreviation	Log Rank P-value	Log Rank FDR	Z-score	Upregulated in:	Deceased Log2 Mean Expression	Living Log2 Mean Expression	T-Test P-value	T-Test FDR
hsa-miR-183-5p	PRAD	7.16e-01	9.34e-01	0.361	Deceased	13.03	12.49	4.65e-01	6.65e-01

Supplementary Figure S2. Association between miR-183 expression and clinical variables in the MSKCC [28] and PRAD-TCGA cohorts. MiRNA expression (RPM) and the indicated tissue or clinical variables of the samples were analyzed by Mann-Whitney test to assess the significance of the differences between the groups and the resulting *p* -values are shown for each comparison. Low/Shorter and High/Longer indicate the group of samples at the 25 and 75 percentiles of the variable distribution respectively. A) Scatterplots of sample values for the indicated clinical variables. B) Table of patient survival dependence on miR-183 expression yielded by OncomiR tool [29].

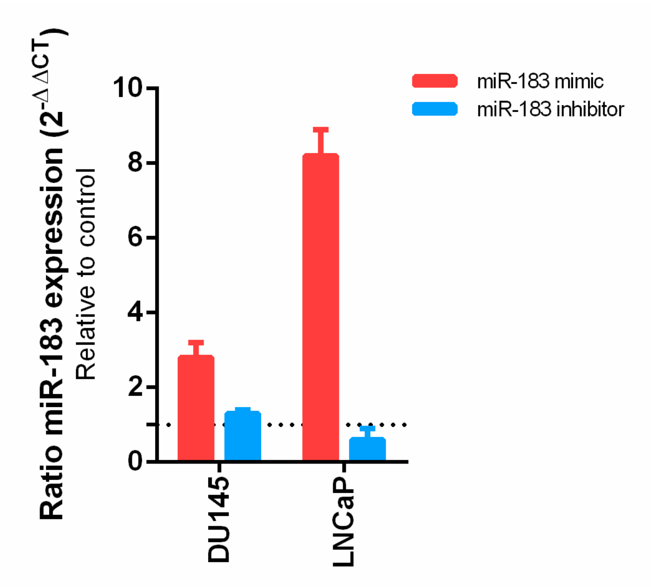

Supplementary Figure S3. MiR-183 expression in the samples used for gene expression microarray experiments. MiR-183 level was assessed by qRT-PCR 24 hours after the transient transfection of 50nM of miScript hsa-miR-183-5p Mimic, 50nM of Allstar Negative Control siRNA or 500nM miScript hsa-miR-183-5p inhibitor (Qiagen) in the indicated cell lines. MiR-183 expression was normalized with RNAU6 and SCARNA17 RNAs ($2^{-\Delta\Delta CT}$) and compared to Allstar Negative Control siRNA transfected cells. The results are shown as mean ± standard deviation of two technical replicates per cell line.

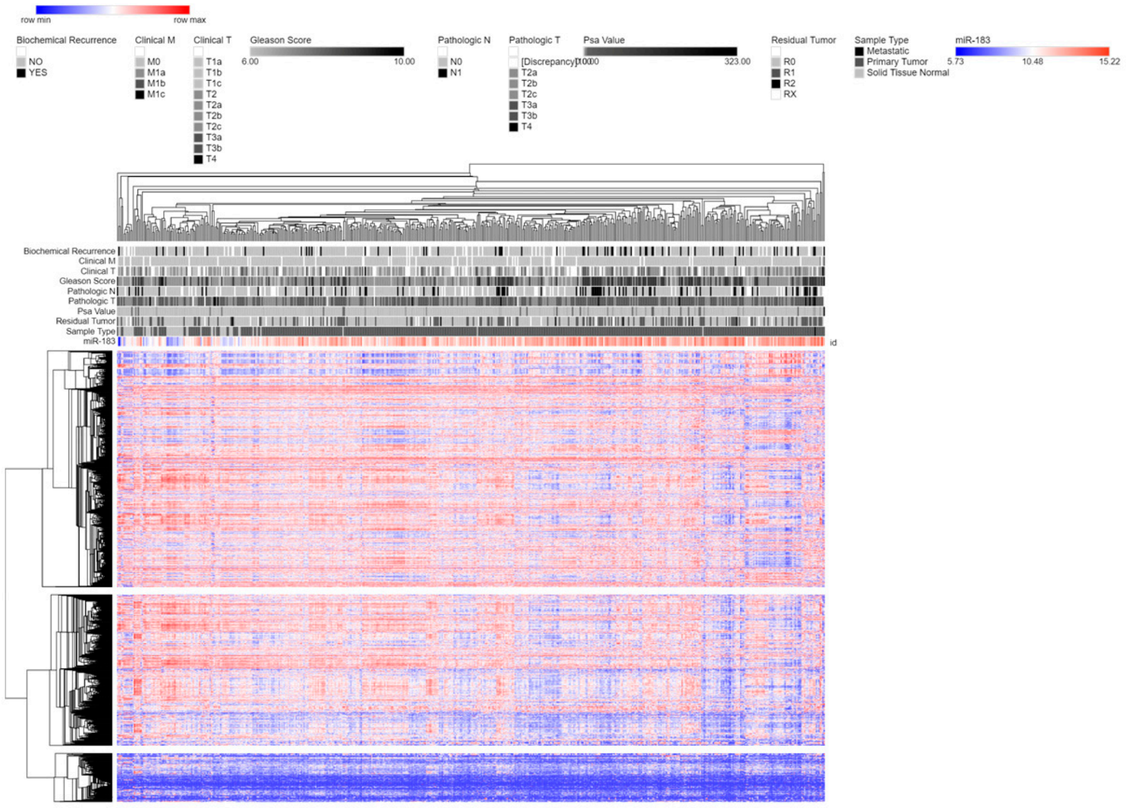

Supplementary Figure S4. Hierarchical clustering of PRAD-TCGA samples based on the expression of miR-183 and cell adhesion genes as well as the status for different clinical parameters. Cell adhesion genes set from the AmiGO repository [70] was selected. Expression of mRNAs and miR-183 and clinical data were downloaded from PRAD-TCGA project using Xena Browser [68]. Euclidean Distance and Spearman rank correlation algorithms for rows and columns, respectively, were applied for clustering using Morpheus. Color scale indicates the relative expression of genes across the samples (red and blue represent higher and lower expression relative to the mean respectively). Status for different clinical parameters (Biochemical Recurrence, Clinical M, Clinical T, Gleason Score, Pathologic N, Pathologic T, PSA Value, Residual Tumor, and Sample Type) is represented in grayscale. The clinical values are indicated in the legends. Horizontal color scale bars indicate the range of the variables.

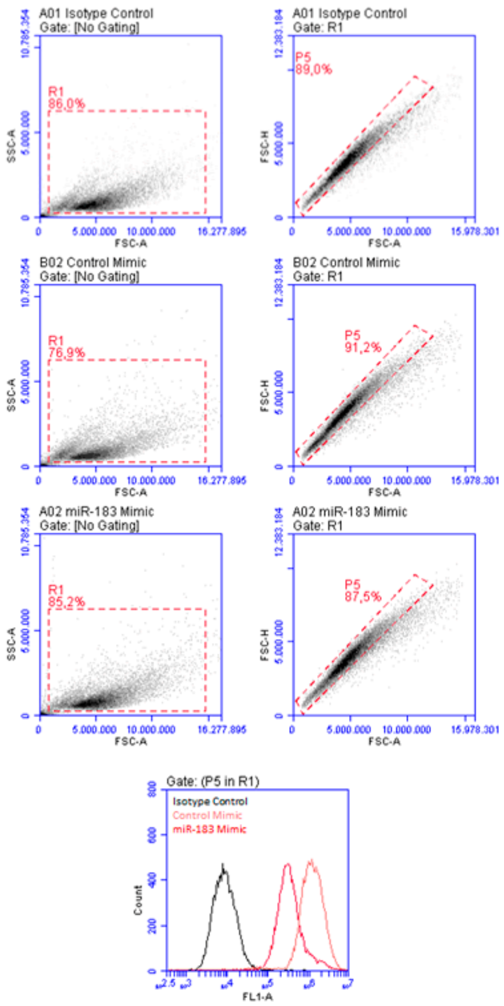

Supplementary Figure S5. Flow-Cytometry analysis of DU145 cells for quantification of ITGB1 protein. Graphs of SSC-A vs. FSC-A, FSC-H vs. FSC-A and Counts vs. FL1-A from untransfected cells incubated with only the secondary IgG antibody (A-11029 Invitrogen) (Isotype Control) or transfected with 20nM of miR-183 or control RNA and incubated with both primary ITGB1 (ab24693 Abcam) and secondary IgG antibody (A-11029 Invitrogen) from a representative experiment in DU145 are shown.

A B

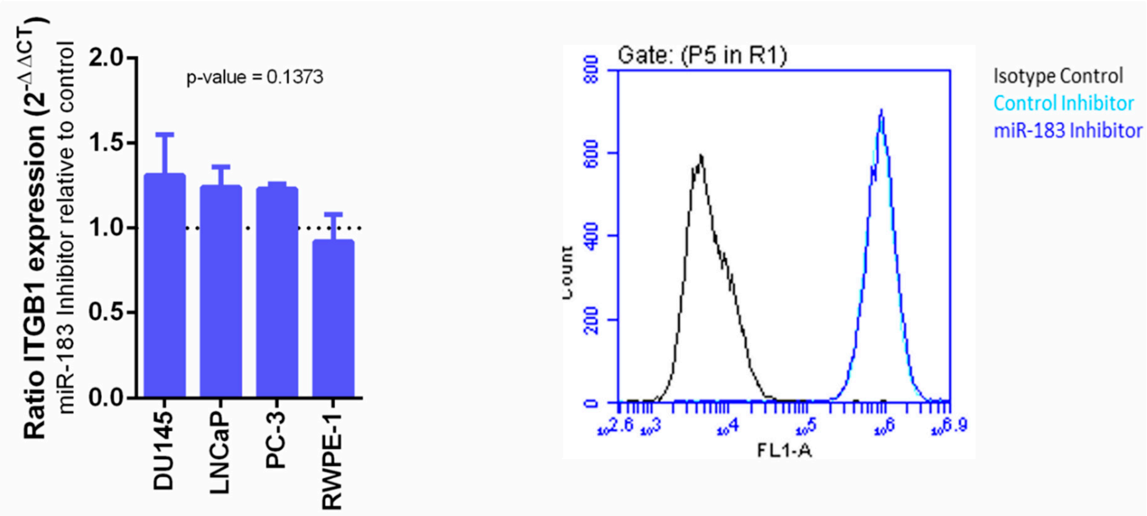

Supplementary Figure S6. Regulation of ITGB1 expression by miR-183 inhibitors. Exponentially growing PrCa cell lines were transfected with 200***μ***M of miR-183 inhibitor or a control RNA and 72 hours afterwards ITGB1 RNA and protein level were analyzed. (A) Relative expression of ITGB1 mRNA in the miR-183 inhibitor or control RNA transfectants. GAPDH and BACT expression was used as a loading normalizer. Two technical replicates were performed per cell line. Average and standard deviation are shown in columns and error bars respectively. Paired t-test was performed considering the 4 cell lines indicated as biological replicates to assess the statistical significance between conditions. (B) Flow-Cytometry analysis of DU145 living cells for quantification of ITGB1 protein (primary ITGB1 antibody (ab24693 Abcam) and secondary IgG antibody (A-11029 Invitrogen)). Counts vs. FL1-A graph from a representative experiment in DU145 is shown.

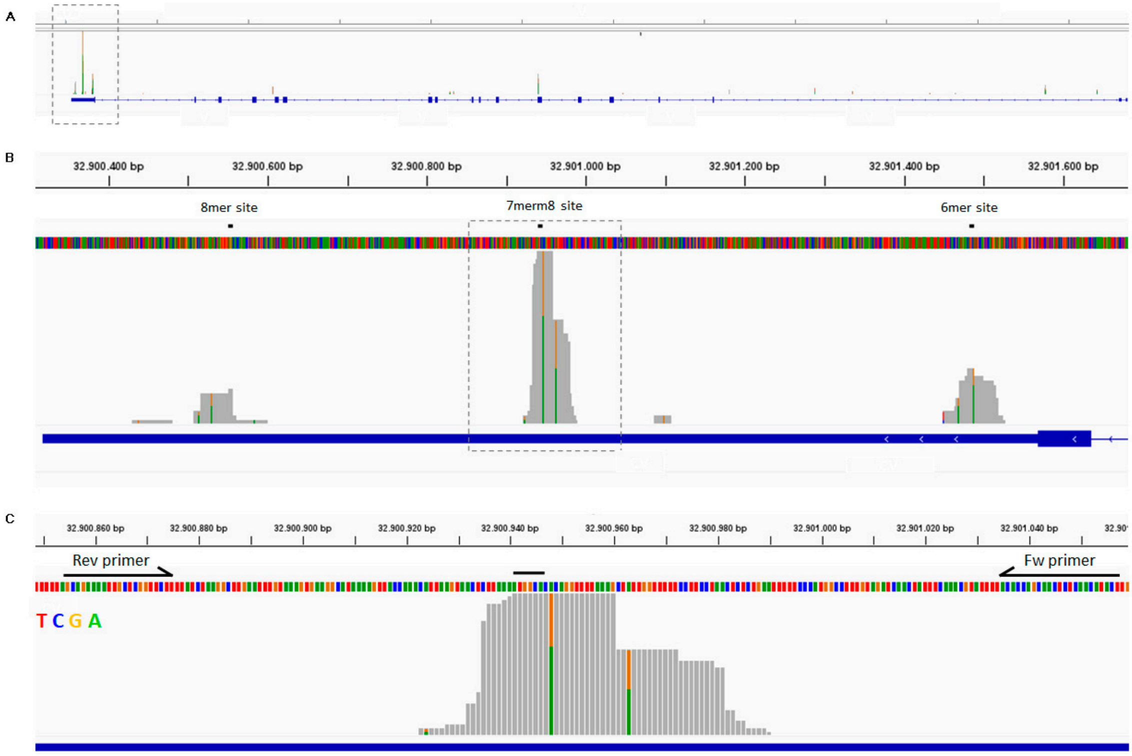

Supplementary Figure S7. Mapping of prostate cell lines AGO-PAR-CLIP reads extracted from Hamilton et al. [31] along the entire ITGB1 gene (A), its 3′ UTR (B) and its vertebrate conserved miR-183 putative binding site (C). Dotted boxes show the region zoomed in the next image. Exons and Introns are represented by blue boxes and lines, respectively. The DNA sequence of the sense strand is shown, but because of the ITGB1 gene orientation in the reference genome, the sequence of the ITGB1 transcript is the complementary. Nucleotide positions with perfect matches and base substitutions caused by the photoactivatable ribonucleoside crosslinking, are represented as gray and color bars in the mapped reads, respectively. The green colored A bar indicates there was no nucleotide substitution, whereas the T-C transitions are shown as orange G bars in the piled reads. Forward and reverse primers used to clone the miR-183 binding site of ITGB1 3′UTR in pmiRGLO are also shown. The image is a modified screenshot of IGV Software [42].

Supplementary Table S1. List of mRNA transcripts repressed by miR-183 identified by gene expression Affymetrix microarrays (Fold change ≥ 1.25). DU145 and LNCaP RNA was analyzed 24 hours after the transient overexpression or inhibition of miR-183. Genes differentially expressed in comparison to the control RNA in at least two conditions were selected.

Supplementary Table S2. List of mRNA transcripts immunoprecipitated with AGO2 antibody that map on conserved canonical miR-183 seeds (6mer and longer) in prostate cell lines. Argonaut-associated transcripts containing motifs perfectly complementary with the 6-mer (2-7 nt) miR-183 seed on AGO-PAR-CLIP experiments of 5 prostate cell lines published by Hamilton et al. [31]. Transcripts appearing in at least 3 cell lines were selected.

Supplementary Table S3. List of mRNA transcripts negatively correlated with miR-183 in PRAD-TCGA samples. Spearman r^2 and *p* -values are shown for each transcript.

Supplementary Table S4. List of the 135 direct candidate target genes of miR-183 resulting of the intersection of selected genes of Supplementary Tables 1-3.

Supplementary Table S5. List of cell adhesion annotated genes from AMIGO2 GO repository [70].

Supplementary Table S6. Matrix of normalized RNA-seq data of the cell adhesion-annotated genes (RPKM) and miR-183 (RPM) as well as the status for different clinical parameters from PRAD-TCGA project used for Hierarchical Clusterization using Morpheus.

Supplementary Table S7. List of miR-183 directed targets published in PrCa indicating their compliance to the criteria used to select direct targets in our study.
